# Hypoxia and aerobic metabolism adaptations of human endothelial cells

**DOI:** 10.1007/s00424-017-1935-9

**Published:** 2017-02-08

**Authors:** Agnieszka Koziel, Wieslawa Jarmuszkiewicz

**Affiliations:** Department of Bioenergetics, Adam Mickiewicz University, Umultowska 89, 61-614 Poznan, Poland

**Keywords:** Mitochondria, Hypoxia, Endothelial cells, Oxidative metabolism, Mitochondrial respiration

## Abstract

The goal of our study was to assess the influence of chronic exposure to hypoxia on mitochondrial oxidative metabolism in human umbilical vein endothelial cells (EA.hy926 line) cultured for 6 days at 1% O_2_ tension. The hypoxia-induced effects were elucidated at the cellular and isolated mitochondria levels. Hypoxia elevated fermentation but did not change mitochondrial biogenesis or the aerobic respiratory capacity of endothelial cells. In endothelial cells, hypoxia caused a general decrease in mitochondrial respiration during carbohydrate, fatty acid, and amino acid oxidation but increased exclusively ketogenic amino acid oxidation. Hypoxia induced an elevation of intracellular and mitochondrial reactive oxygen species (ROS) formation, although cell viability was unchanged and antioxidant systems (superoxide dismutases SOD1 and SOD2, and uncoupling proteins (UCPs)) were not increased. In mitochondria from hypoxic cells, the opposite change was observed at the respiratory chain level, i.e., considerably elevated expression and activity of complex II, and decreased expression and activity of complex I were observed. The elevated activity of complex II resulted in an increase in succinate-sustained mitochondrial ROS formation, mainly through increased reverse electron transport. A hypoxia-induced decrease in UCP2 expression and activity was also observed. It can be concluded that the exposure to chronic hypoxia induces a shift from aerobic toward anaerobic catabolic metabolism. The hypoxia-induced increase in intracellular and mitochondrial ROS formation was not excessive and may be involved in endothelial signaling of hypoxic responses. Our results indicate an important role of succinate, complex II, and reverse electron transport in hypoxia-induced adjustments in endothelial cells.

## Introduction

As the first cell layer in contact with blood, endothelial cells have to cope with various physiological and pathophysiological changes within the blood components. One challenging stimulus is deficiency in the oxygen tension. The response of endothelial cells to hypoxic stress can have two different consequences in the surrounding tissues, depending on the duration of the exposure: (i) short-term exposure causes physiological and reversible modulation of vascular tone and blood flow, and (ii) chronic hypoxic stress results in irreversible remodeling of the vasculature and surrounding tissues with smooth muscle proliferation and fibrosis [8]. Persistent hypoxia occurs frequently in many disorders, such as chronic obstructive pulmonary disease, pulmonary hypertension, obstructive sleep apnea, diffuse interstitial fibrosis, atherosclerosis, sickle cell disease, and systemic sclerosis [8, 29]. Hypoxia is also observed in ischemia and cancer. In endothelial cells, hypoxia initiates a number of responses that include cell growth and proliferation, increased permeability, and changes in cell-surface adhesion molecules. The two endothelium-dependent pathways involving cyclooxygenase (COX) and endothelium nitric oxide synthase (eNOS) are activated in hypoxic augmentation. Exposure of pulmonary artery endothelial cells to hypoxia triggers a retrograde mitochondrial movement, resulting in the perinuclear clustering of mitochondria accompanied by the accumulation of reactive oxygen species (ROS) in the nucleus [27]. Endothelial mitochondria may act as oxygen sensors in the hypoxic response signal cascade and produce more mitochondrial ROS (mROS), which are important signaling molecules in vascular endothelial cells [2, 30]. NO produced by endothelial cells can regulate the activity of hypoxia-inducible factor 1-alpha (HIF1-α) and 5′AMP-activated protein kinase (AMPK), affecting key hypoxia and metabolic stress response pathways, respectively [24]. It remains unclear how mROS production in endothelial cells triggers ROS production from other cellular sources and activates AMPK, which can alter fuel selectivity and protect cells from apoptosis [4, 24]. Some studies suggest that endothelial mitochondria regulate HIF-1α and HIF-2α stabilization by releasing mROS to the cytosol and subsequently restrain ROS production in chronic hypoxia to avoid cellular damage [4]. In endothelial cells, mitochondria are central mediators of intrinsic apoptosis, which is initiated by cellular stressors, including hypoxia [27]. It has also been proposed that endothelial mitochondria may sense hypoxia and transmit, via mROS, a signal to the endoplasmic reticulum [18] that in turn releases Ca^2+^ [23]. In pulmonary artery hypertension, mitochondrial signaling modulates the chronic response of the pulmonary circulation to hypoxia. Decrease in mitochondrial glucose oxidation provides several advantages to endothelial cells, including diversion of pyruvate into anabolic pathways, suppression of apoptosis, promotion of cell proliferation, and activation of the HIF-1, which increases pyruvate dehydrogenase kinase (PDK) expression, thus sustaining mitochondrial suppression [5].

The influence of hypoxic exposure of endothelial cells, especially chronic exposure to hypoxia, on mitochondrial oxidative function has not been intensively studied. Many questions must be addressed with respect to understanding the role of endothelial mitochondria in response to hypoxia-induced metabolic disturbances that may lead to the development of vascular dysfunction and atherosclerosis. Thus, the aim of the present study was to elucidate the effects of a chronic 6-day hypoxic exposure of cultured human endothelial EA.hy926 cells to 1% O_2_ on aerobic metabolism at the cellular and mitochondrial levels. Cell viability, superoxide formation, and mitochondrial respiratory function, including the respiratory response to different reducing fuels and mitochondrial oxidative capacity, were monitored in hypoxic cells. Moreover, we examined the effect of chronic exposure of growing endothelial cells to hypoxia on mitochondria by measuring their respiratory activities with complex I and complex II substrates, ATP synthesis, mitochondrial membrane potential (mΔΨ), mitochondrial uncoupling protein (UCP)-mediated uncoupling, and mROS formation.

## Materials and methods

### Cell culture and cell fraction preparation

We used the human endothelial cell line EA.hy926 (ATCC® CRL-2922™), which was originally derived from a human umbilical vein [7]. Cells were grown in Dulbecco’s modified Eagle’s medium (DMEM) supplemented with 10% fetal bovine serum (FBS), 1% L-glutamine, 2% hypoxanthine-aminopterin-thymidine (HAT), and 1% penicillin/streptomycin. The EA.hy926 cells were cultured for 6 days in a humidified 5% CO_2_ atmosphere at 37 °C at 20% O_2_ (normoxic conditions) or 1% O_2_ (hypoxic conditions). Cells were cultured in 140-mm dishes until they reached approximately 90–100% confluence. Cells that were used in this study were between passages 5 and 12.

EA.hy926 cells from both the control and hypoxic cultures were harvested with trypsin/EDTA, rinsed twice with phosphate-buffered saline (PBS) (containing 10 or 5% FBS), and centrifuged at 1200*g* for 10 min. Subsequently, the cells were washed in cold PBS and then centrifuged again. The final cell pellet was resuspended in PBS (1 g of cells per 2 ml of medium) and maintained on ice. Protein content was determined using the Bradford method (Bio-Rad). The yield of harvested cells differed significantly between the control and the hypoxia-treated cells. Namely, 4.3 ± 0.6 and 3.8 ± 0.05 g of cells (SD, *n* = 25, *P* < 0.05) were harvested from 50 dishes of control and hypoxia-exposed cells, respectively (when cells were inoculated at the same density).

### Measurements of cell respiration

The detached control and hypoxia-treated EA.hy926 cells were resuspended in cold DMEJ medium, instead of PBS medium, containing 5.4 mM KCl, 0.8 mM MgSO_4_, 110 mM NaCl, 44 mM NaHCO_3_, 1.1 mM NaH_2_PO_4_, and 10 mM Na/Na buffer (pH 7.2). Oxygen consumption rate (OCR) was measured at 37 °C using a Clark-type electrode (Hansatech) in 0.7 ml of DMEJ medium with 2–3 mg of protein.

The mitochondrial function in detached EA.hy926 cells was determined polarographically as previously described [15]. The following respiratory substrates were used: 5 mM pyruvate, 5.5 mM glucose, 5 mM glutamine, 0.3 mM palmitic acid, 5 mM lysine, 5 mm leucine, 5 mM valine, or 5 mM threonine. To estimate the contribution of ATP-linked OCR and non-ATP-linked OCR (proton leak) to the basal respiratory rate, oligomycin (1 μg/ml) was added to inhibit ATP synthesis. Subsequently, the proton ionophore (uncoupler) carbonyl cyanide 4-(trifluoromethoxy)phenylhydrazone (FCCP, 0.5 μM) was added to determine the maximal oxygen uptake that the cells could sustain. Finally, cyanide (0.5 mM) was added to inhibit complex IV (cytochrome *c* oxidase, COX) and thereby block the entire mitochondrial cytochrome pathway. In the presence of cyanide, no residual (non-mitochondrial) respiration was observed.

### Mitochondrial isolation and cytosolic fraction preparation

Mitochondria were isolated from EA.hy926 cells using a very efficient isolation procedure that produces highly active and well-coupled mitochondria [15]. The yields of the isolated mitochondria were equal to 3.4 ± 0.3 and 3.1 ± 0.3 mg of mitochondrial protein per gram of cells (SD, *n* = 15, *P* < 0.05) for cells grown under control or hypoxic conditions, respectively.

To obtain cytosolic fractions for enzymatic measurements, cells were homogenized in one step in PREPII medium with the Polytron homogenizer (T18 basic, IKA) (eight times for 5 s, at 80% power). The homogenates were subsequently centrifuged at 1200*g* for 10 min. After spinning down the unbroken cells and cell debris, the supernatants were collected for measuring the activities of citrate synthase (CS), COX, and lactate dehydrogenase (LDH).

### Measurements of mitochondrial respiration and membrane potential

Mitochondrial respiration and membrane potential (mΔΨ) were measured in isolated endothelial mitochondria as previously described [1, 15]. Oxygen uptake was determined polarographically using a Rank Bros. (Cambridge, UK) oxygen electrode or a Hansatech oxygen electrode in either 0.7 or 2.8 ml of standard incubation medium (at 37 °C), which consisted of 150 mM sucrose, 2.5 mM KH_2_PO_4_, 2 mM MgCl_2_, 1.5 mM EGTA, 20 mM Tris/HCl (pH 7.2), and 0.1% BSA, with either 0.5 or 2 mg of mitochondrial protein (0.7 mg of mitochondrial protein per 1 ml). O_2_ uptake values are presented in nanomole O_2_ × min^−1^ × mg^−1^ protein. Membrane potential was measured simultaneously with oxygen uptake using a tetraphenylphosphonium (TPP^+^)-specific electrode. The values for mΔΨ are given in millivolts. The 5 mM TCA substrates (malate, succinate in the presence or absence of 2 μM rotenone, pyruvate, α-ketoglutarate, and isocitrate), 5 mM glutamate, and 0.3 mM palmitoylcarnitine were used as respiratory substrates.

Phosphorylating respiration was measured using 150 μM ADP (pulse), and uncoupled respiration was measured using up to 0.5 μM FCCP. Only high-quality mitochondria preparations, i.e., those with an ADP/O value of approximately 2.3 and a respiratory control ratio (RCR) of approximately 3.6–4.2 (with malate as a respiratory substrate), were used in the experiments. Non-phosphorylating (resting state, state 4) respiration measurements were performed in the absence of exogenous ADP.

The proton leak UCP-mediated measurements were performed with 5 mM succinate (plus 2 μM rotenone) as an oxidizable substrate, in the presence of 1.8 μM carboxyatractyloside and 0.5 μg/ml oligomycin, which inhibit the activities of the ATP/ADP antiporter and ATP synthase, respectively. The response of proton conductance to its driving force can be expressed as the relationship between the oxygen consumption rate and the mΔΨ (flux-force relationship) when varying the potential via titration with respiratory chain inhibitors. To decrease the rate of the coenzyme QH_2_-oxidizing pathway, succinate dehydrogenase was titrated with cyanide (up to 20 μM). To induce UCP activity, 14 μM linoleic acid or 100 μM 4-hydroxy-2-nonenal (HNE) was used. HNE was added to the mitochondria 15 min before the TPP^+^ calibration and the mitochondrial energization with succinate. To inhibit UCP activity, 4 mM GTP was applied.

### Measurement of enzyme activities

The activity of CS was determined by tracking the formation of DTNB-CoA at 412 nm using a UV 1620 Shimadzu spectrophotometer as described previously [15]. The reaction mixture contained 100 mM Tris/HCl (pH 8.0) 100 μM acetyl CoA, 100 μM 5,5′-di-thiobis-(2-nitrobenzoic acid) (DTNB), 0.1% Triton X-100, and 100 μM oxaloacetate. The activity of LDH was measured by spectrophotometer at 340 nm by following the oxidation of NADH (150 μM) mixed with pyruvate (10 mM) in 50 mM Tris/HCl (pH 7.3). The activity of both enzymes was measured in 50 μg of protein from the cytosolic fractions.

The maximal activity of COX and the integrity of the outer mitochondrial membrane were assessed polarographically as described previously [15].

All enzymatic measurements were performed at 37 °C with continuous stirring.

### Determination of superoxide anion formation in cells

ROS production was detected using a nitroblue tetrazolium (NBT) assay with normoxic and hypoxic EA.hy926 cells. NBT (yellow water soluble) was reduced by superoxide to formazan-NBT (dark-blue water insoluble). The assay was performed by incubating detached cells (0.2 mg of protein in 1 ml DMEM medium) with 0.2% NBT under agitation. The samples were incubated for 1 h (37 °C) in the presence or absence of 10 μM diphenylene iodonium (DPI) (an inhibitor of NADPH oxidase and endothelial nitric oxide synthase (eNOS), enzymes involved in endothelial ROS formation). The cells were centrifuged (1200*g* for 10 min at 4 °C), the supernatant was removed, and the formazan-NBT was dissolved in 200 μl of 50% acetic acid by sonication (three pulses of 10 s each; Bandelin Electronic). The samples were briefly centrifuged, and the absorbance of the supernatant was determined at 560 nm using a UV 1620 Shimadzu spectrophotometer.

Additionally, mitochondrial superoxide formation was measured using MitoSOX Red (Invitrogen), a specific fluorescent mitochondrial superoxide indicator. The assay was performed by incubating adherent cells (grown in 96-well plates) with 5 μM MitoSOX in PBS containing 5.5 mM D-glucose and 5 mM pyruvate for 10 min at 37 °C. Cells were washed twice with PBS. Fluorescence emission at 595 nm under 510 nm excitation was recorded using an Infinite M200 PRO Tecan multimode reader.

### Determination of H_2_O_2_ production by isolated endothelial mitochondria

Mitochondrial H_2_O_2_ production was measured by the Amplex Red horseradish peroxidase method (Invitrogen). Horseradish peroxidase (0.1 units/ml) catalyzes the H_2_O_2_-dependent oxidation of non-fluorescent Amplex Red (5 μM) to fluorescent resorufin red. The fluorescence kinetics were followed for 15 min at an excitation wavelength of 545 nm and an emission wavelength of 590 nm using an Infinite M200 PRO Tecan multimode reader. Mitochondria (0.1 mg of mitochondrial protein) were incubated in 0.5 ml of the standard incubation medium (see above) with 5 mM succinate plus or minus 2 μM rotenone, 5 mM malate, or 5 mM succinate and 5 mM malate, in the absence (non-phosphorylating state 4 conditions) or presence of 150 μM ADP (phosphorylating state 3 conditions). Reactions were monitored with constant stirring. H_2_O_2_ production rates were determined by calculating slopes from readings obtained along several 15-min repeated measurements.

### Trypan blue cell viability assay

Both living and dead cells were harvested from cultures. After cell harvesting, 0.4% trypan blue solution was added (1:1 *v*/*v*), and cell viability was determined using a Countess Automated Cell Counter (Invitrogen). In a trypan blue exclusion assay, cells that take up the dye are either necrotic or apoptotic.

### Determination of protein levels via immunoblotting

RIPA buffer (150 mM NaCl, 1% Triton X-100, 0.5% Na deoxycholate, 0.1% SDS, 50 mM Tris (pH 8.0)) was used to lyse endothelial cells. The cellular and mitochondrial fractions were isolated in the presence of protease inhibitors (Sigma). The proteins were separated on an 8–12% SDS-PAGE gel. The Spectra™ Multicolor Broad Range Protein Ladder (Fermentas) was used as a molecular weight marker. The following primary antibodies were used: anti-hypoxia-inducible factor 1-alpha (HIF1-α, 93 kDa) (ab51608, Abcam), anti-hexokinase I (HK I, 120 kDa) (sc-80978, Santa Cruz Biotechnology), lactate dehydrogenase (LDH, 35 kDa) (sc-133123, Santa Cruz Biotechnology), anti-β-actin (42 kDa) (CP01, Calbiochem), anti-superoxidase dismutase 1 (SOD1, 18 kDa) (ab-13498, Abcam), anti-glyceraldehyde 3-phosphate dehydrogenase (GAPDH, 40 kDa) (ab-9485, Abcam), anti-acyl-coenzyme A dehydrogenase (ACADS, 44 kDa) (ab-154823, Abcam), anti-glutamate dehydrogenase (GDH, 61 kDa) (ab89967, Abcam), anti-E3-binding protein of pyruvate dehydrogenase (E3BP, 54 kDa) (sc-79236, Santa Cruz Biotechnology), anti-UCP2 (33 kDa) (ab97931, Abcam), anti-UCP3 (34 kDa) (ab3477, Abcam), anti-mitochondrial superoxide dismutase (SOD2, 25 kDa) (ADI-SOD-110, Enzo Life Sciences), anti-citrate synthase (CS, 52 kDa) (ab-96600, Abcam), anti-mitochondrial marker (MTC02, 60 kDa) (ab3298, Abcam), anti-cytochrome *c* oxidase subunit II (COXII, 26 kDa) (ab-110258), and the MitoProfile® total OXPHOS human antibody cocktail (ab-110411, Abcam) containing antibodies raised against subunits of complex I (20 kDa subunit NDUFB8), complex II (30 kDa subunit), complex III (subunit core 2, 47 kDa), complex IV (COXII, 24 kDa), and ATP synthase (subunit α, 57 kDa). Appropriate horseradish peroxidase-conjugated secondary antibodies were used. The expression levels of COXII or a mitochondrial marker (for the mitochondrial fractions) and of β-actin or GAPDH (for the cell fractions) were used as loading controls for normalization. Protein bands were visualized using the Amersham ECL system and digitally quantified using the GeneTools 4.03 software package.

### Statistical analysis

Data are presented as the means ± SD obtained from at least five to ten independent experiments (cell suspension preparations or mitochondrial isolations), and each determination was performed at least in duplicate. Significant differences were determined via unpaired *t* tests or ANOVAs (followed by Tukey’s post hoc comparisons for *P* < 0.05 from an ANOVA). Differences between cells grown under hypoxic and normoxic conditions were considered statistically significant at *P* < 0.05 (*), *P* < 0.01 (**), or *P* < 0.001 (***).

## Results

### Hypoxia elevated fermentation in endothelial cells but did not change mitochondrial biogenesis or aerobic respiration capacity

**Fig. 1:**
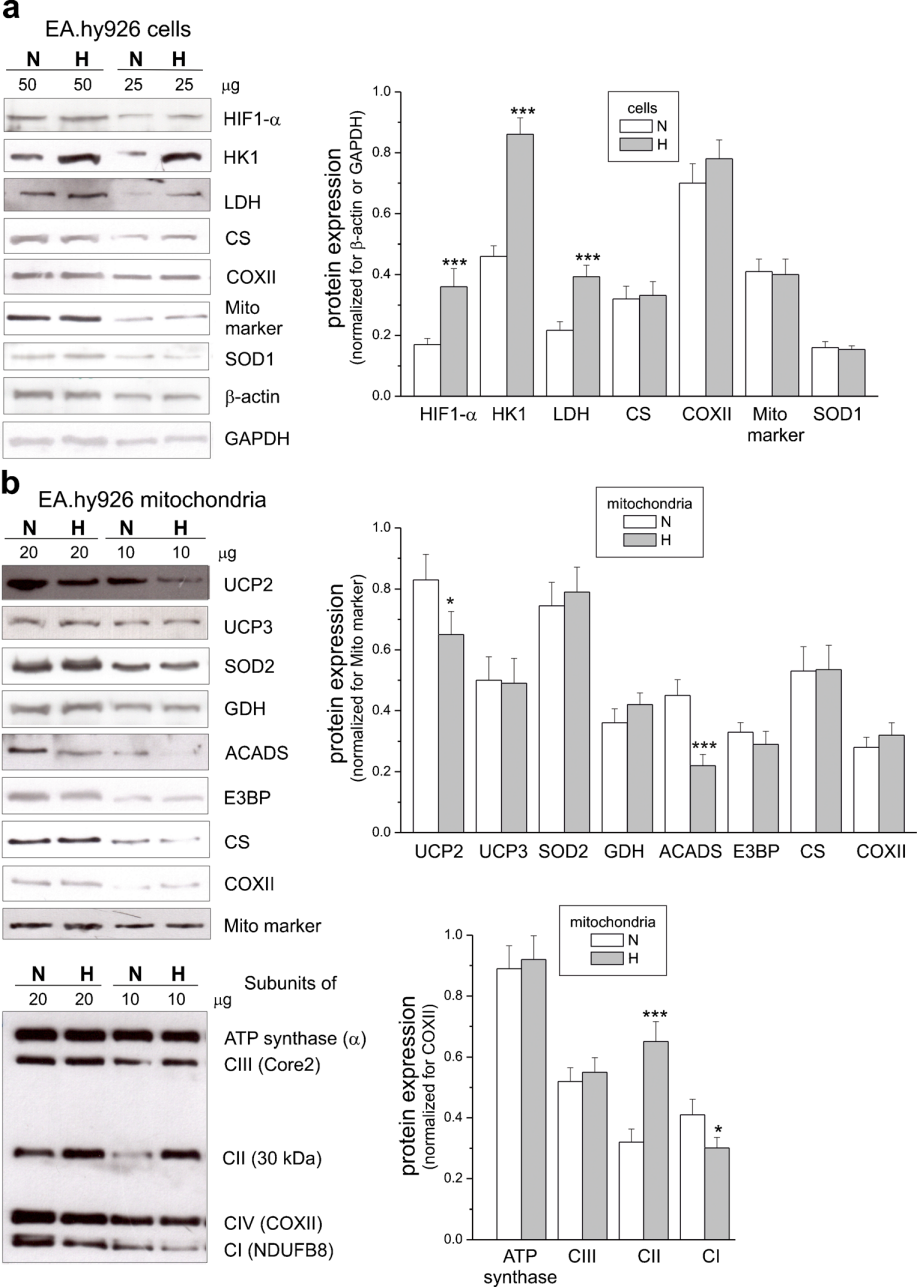
Representative Western blots (*left*) and analyses of the protein expression (*right*) in endothelial cells grown for 6 days under normoxic (*N*) and hypoxic (*H*) conditions (**b**) and in mitochondria isolated from these cells (**a**). *HKI* hexokinase I, *COXII* cytochrome *c* oxidase subunit II, *SOD1* and *SOD2* superoxide dismutases, *UCP2* and *UCP3* uncoupling proteins, *GDH* glutamate dehydrogenase, *ACADS* acyl-coenzyme A dehydrogenase, *E3BP* pyruvate dehydrogenase, *CI-CIV*, complexes of respiratory chain, *Mito marker* mitochondrial marker, *GAPDH* glyceraldehyde 3-phosphate dehydrogenase. Means ± SD; *n* = 10. *P* < 0.05 (*), *P* < 0.001 (***), comparison vs. control values (*N*)

**Fig. 2:**
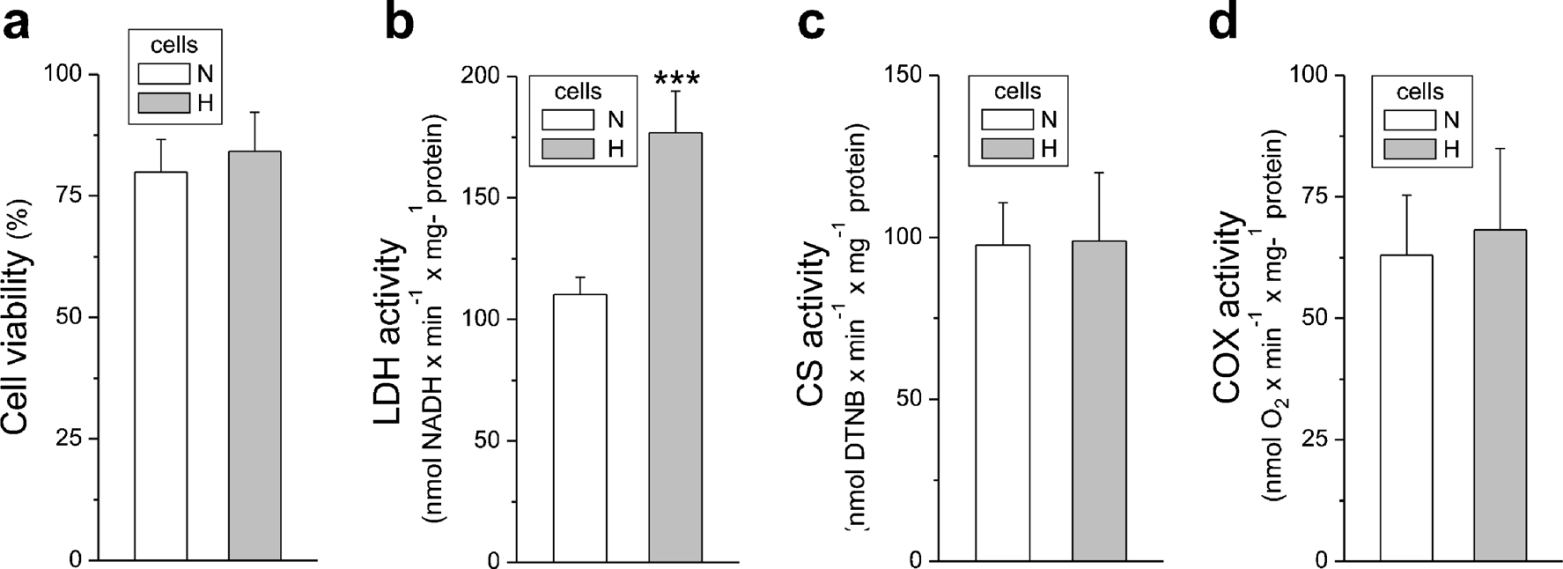
Cell viability (**a**) and maximal activities of marker enzymes of aerobic (**c**, **d**) and anaerobic (**b**) catabolism of EA.hy926 cells grown in normoxia (*N*) or chronic hypoxia (*H*). Means ± SD; *n* = 8. *P* < 0.001 (***), comparison vs. control values (*N*)

In endothelial EA.hy926 cells, exposure to chronic hypoxia for 6 days significantly elevated the expression levels of hexokinase I, the enzyme catalyzing the first rate-limiting step of the glycolytic pathway, and LDH, the enzyme that catalyzes the interconversion of pyruvate and lactate, and levels of HIF1-α were increased (Fig. 1a). These changes were accompanied by a considerable ∼60% increase in LDH activity (Fig. 2b), indicating that cells grown in hypoxia display intensified anaerobic glucose oxidation via the glycolytic pathway and lactic acid fermentation. Notably, cell viability was unaltered in chronically hypoxic cells compared to normoxic cells (Fig. 2a).

The endothelial cells cultured under normoxic and hypoxic conditions exhibited similar activities (Fig. 2c, d) and expression levels (Fig. 1a) of CS and COX, indicating no change in the capacities of the TCA cycle or the mitochondrial respiratory chain and unaltered mitochondrial biogenesis (mitochondrial content).

### Hypoxia caused a general decrease in mitochondrial respiration except for increased ketogenic amino acid oxidation in endothelial cells

**Fig. 3:**
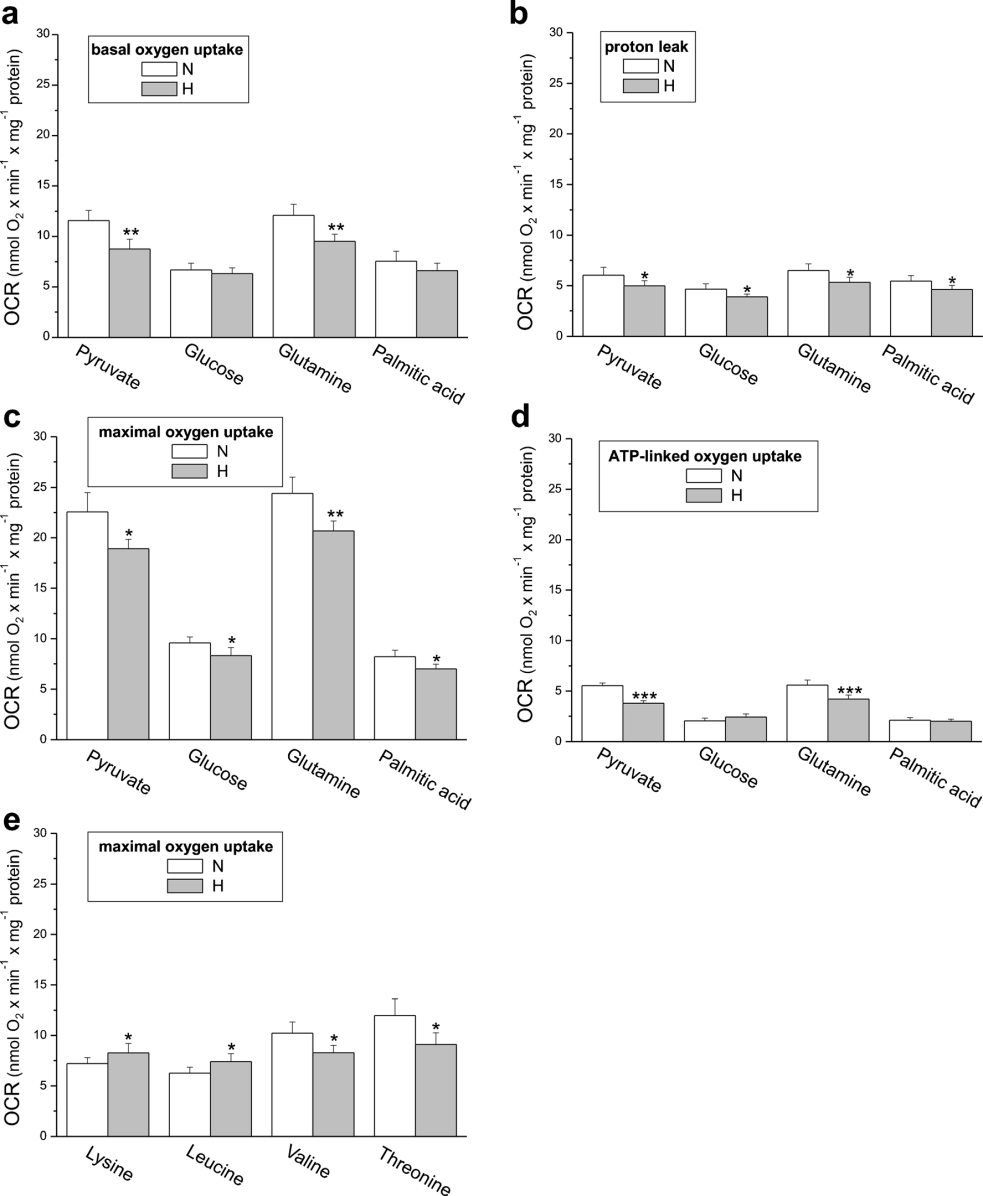
Oxidative metabolism of EA.hy926 cells grown in normoxia (*N*) or chronic hypoxia (*H*). Substrate-dependent changes in the basal oxygen consumption rate (*OCR*) (**a**), proton leak (**b**), maximal oxygen uptake (**c**, **e**), and ATP-dependent oxygen uptake (**d**). Means ± SD; *n* = 6. *P* < 0.05 (*), *P* < 0.01 (**), *P* < 0.001 (***), comparison vs. control values (*N*)

Under basal conditions (basal OCR) (Fig. 3a), carbonyl cyanide-*p*-trifluoromethoxyphenyl-hydrazone (FCCP)-stimulated conditions (maximal OCR) (Fig. 3c) and in the presence of oligomycin (oligomycin-resistant, ATP-linked OCR) (Fig. 3d), both types of cells demonstrated the highest OCR with pyruvate or glutamine. The non-ATP-linked OCR (proton leak) exhibited the least dependence on the type of reducing substrate present (Fig. 3c).

We determined how aerobic metabolism in endothelial EA.hy926 cells supplied with different reducing fuels was altered by long-term cell growth at 1% O_2_. In general, under all respiratory conditions and with respect to all reducing fuels except the exclusively ketogenic amino acids lysine and leucine, hypoxic cells displayed reduced mitochondrial function (Fig. 3). In particular, cells exposed to hypoxia exhibited ∼13–25% reductions in their maximal mitochondrial respiratory capacity in the presence of pyruvate, glucose, palmitic acid, or amino acids (glutamine, valine, and threonine) (Fig. 3c, e). Similarly, with pyruvate and glutamine, a significantly reduced ATP-linked OCR was observed in hypoxic cells, indicating diminished levels of mitochondrial oxidative phosphorylation (Fig. 3d). Interestingly, a significant reduction of non-ATP-linked OCR was also found during oxidation of all substrates (Fig. 3b), indicating decreased proton leak in hypoxic cells. Thus, mitochondrial respiratory measurements indicate a general reduction in mitochondrial respiration during carbohydrate, fatty acid, and glucogenic amino acid oxidation in hypoxia-exposed endothelial cells. In contrast, the maximal oxidation of lysine and leucine was significantly higher in hypoxic cells (Fig. 3e), indicating a greater contribution from exclusively ketogenic amino acids as a fuel source for endothelial respiration during growth under hypoxic conditions.

### Growth in hypoxic conditions increased mitochondrial and non-mitochondrial superoxide generation in endothelial cells

**Fig. 4:**
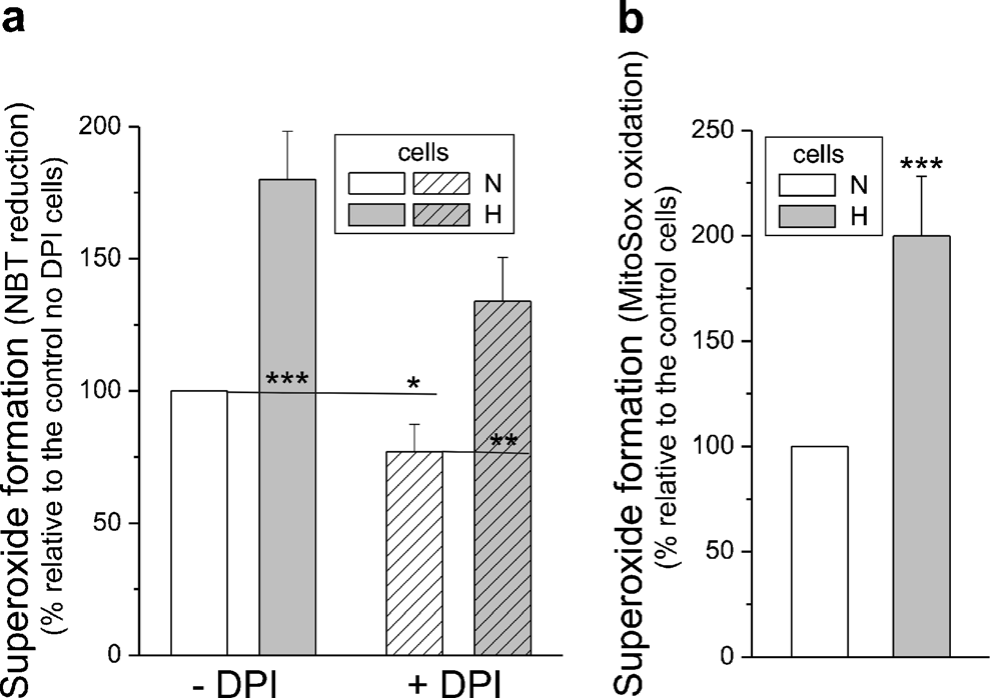
Cellular and mitochondrial superoxide formation in endothelial cells grown in normoxia (*N*) or chronic hypoxia (*H*). Measurements of superoxide formation with nitroblue tetrazolium (*NBT*) in the absence or presence of diphenylene iodonium (*DPI*) (**a**) and with the MitoSOX probe (**b**). Means ± SD; *n* = 7. *P* < 0.05 (*), *P* < 0.01 (**), *P* < 0.001 (***), comparison vs. control values (*N*)

Compared with the normoxic cells, the exposure of EA.hy926 cells to chronic hypoxia caused a significant elevation in total (∼80%) and mitochondrial (∼75–100%) superoxide generation (Fig. 4). Mitochondrial superoxide generation was measured either as DPI-insensitive NBT reduction (Fig. 4a) or MitoSOX oxidation (Fig. 4b). We found that in hypoxic cells, DPI, a flavoprotein inhibitor of NADPH oxidase and eNOS, significantly inhibited (∼30%) hypoxia-induced superoxide generation (Fig. 4a). Thus, in endothelial cells, hypoxia-induced ROS production appears to occur via enzymes and mitochondria (including DPI-insensitive respiratory chain sources).

### Hypoxia-induced changes in the endothelial mitochondrial oxidative phosphorylation system: opposite changes in complex II (upregulation) and complex I (downregulation)

**Fig. 5:**
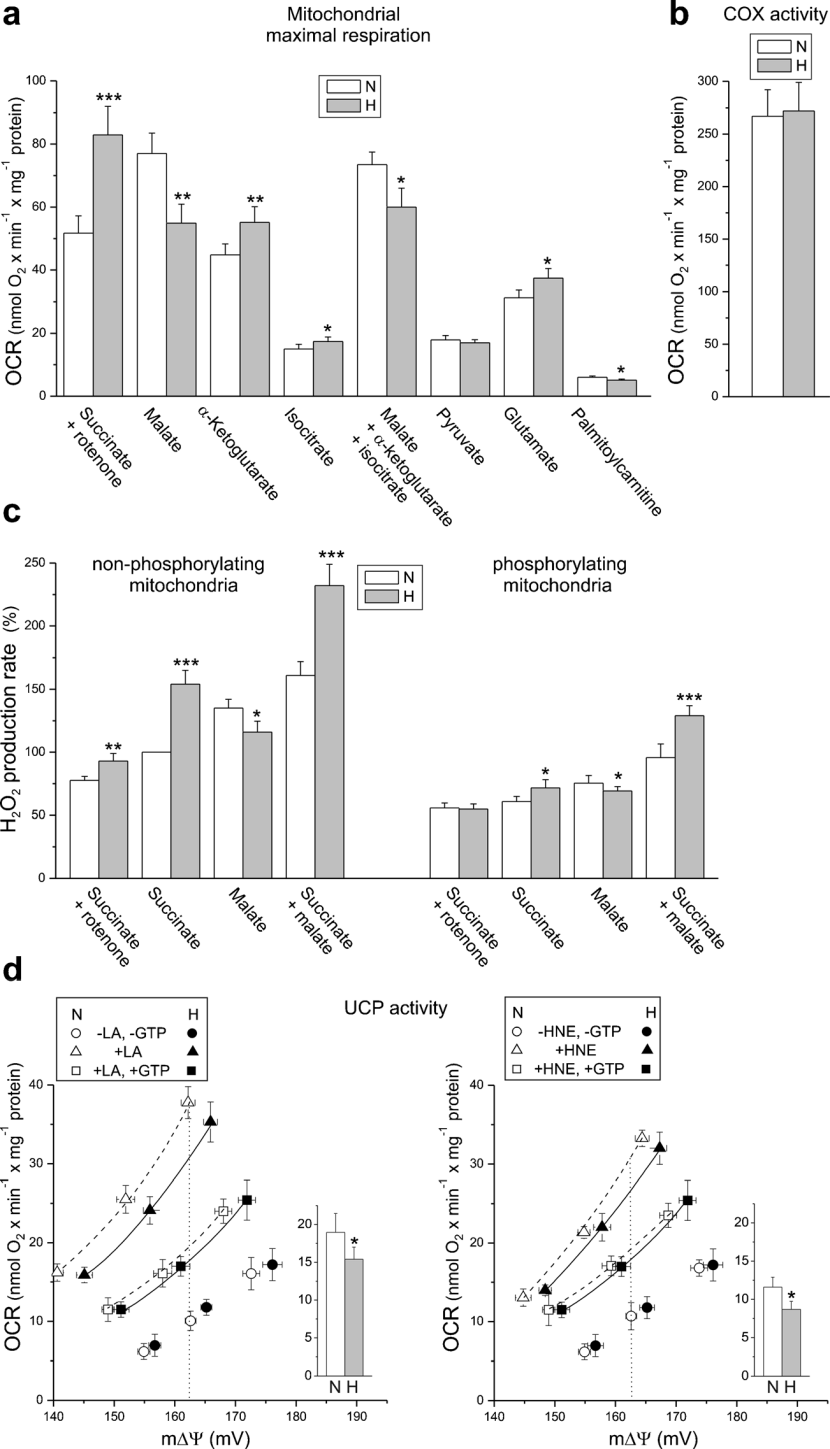
Functional characteristics of endothelial mitochondria isolated from cells grown in normoxia (*N*) or chronic hypoxia (*H*). Maximal (phosphorylating or uncoupled) respiration with various respiratory substrates (**a**), COX activity (**b**), and H_2_O_2_ production with various substrates (**c**). Percentage relative to the control (*N*) mitochondria oxidizing succinate alone (100%). The linoleic acid (*LA*)-induced (**d**, *left*) or HNE-induced (**d**, *right*) GTP-inhibited UCP activity. The relationship between the respiratory rate and mΔΨ (proton leak kinetics) during non-phosphorylating succinate oxidation titrated with cyanide. The LA-induced (*left inset*) or HNE-induced (*right inset*), GTP-inhibited UCP-mediated proton leak (in nmol O_2_ × min^−1^ × mg^−1^ protein) at the same mΔΨ (162 mV). Means ± SD; *n* = 4–8. *P* < 0.05 (*), *P* < 0.01 (**), *P* < 0.001 (***), comparison vs. control values (*N*)

To determine the effect of hypoxia on respiratory activity at the mitochondrial level, we measured the maximal respiratory rate with various reducing substrates in isolated endothelial mitochondria (Fig. 5a). In mitochondria from normoxic cells, the highest maximal respiration was observed with malate or a mixture of NAD-linked substrates (malate, α-ketoglutarate, and isocitrate), which saturate the capacity of the endothelial respiratory chain. In mitochondria from hypoxic cells, a significant decrease (∼30%) in malate-sustained complex I activity (Fig. 5a) accompanied by a ∼30% reduction in the expression level of complex I NDUFB8 subunit (Fig. 1b) was found. In addition, respiration with the mixture of NAD-linked substrates was also reduced by ∼20% (Fig. 5a). Interestingly, in mitochondria from hypoxic cells, the maximal oxidation of α-ketoglutarate alone or isocitrate alone was significantly increased but did not exceed malate oxidation, which seems to saturate the decreased capacity of complex I. In mitochondria from hypoxic endothelial cells, the reduced activity of complex I was revealed by significantly decreased respiratory rates and mΔΨ during phosphorylating and non-phosphorylating oxidation of malate (Table 1). In contrast to the reduction of activity and expression of complex I, the exposure of endothelial cells to hypoxia caused a significant increase (∼60%) in succinate oxidation in mitochondria (Fig. 5a) accompanied by a ∼100% elevation of the complex II expression level (Fig. 1b). In mitochondria from hypoxic cells, the elevated activity of complex II was revealed by significantly increased respiratory rates and mΔΨ during phosphorylating and non-phosphorylating oxidation of succinate in the presence of rotenone (Table 1). Despite changes in activities of complex I and complex II, the efficiency of oxidative phosphorylation (ADP/O ratio) during oxidation of malate or succinate was not significantly changed, although a slight decreasing or increasing tendency, respectively, was observed.

**Table 1:** Respiratory rates, mΔΨ values, and coupling parameters in mitochondria isolated from endothelial EA.hy926 cells cultured under normoxic (N) and hypoxic (H) conditions

	Succinate + rotenone		Malate	
	N	H	N	H
State 3 rate	51.8 ± 4.8	↑ 82.9 ± 5.4***	74.0 ± 7.0	↓ 52.5 ± 4.2*
State 3 mΔΨ	152.3 ± 0.7	↑ 160.4 ± 1.1**	161.3 ± 1.2	↓ 152.6 ± 0.9**
State 4 rate	19.6 ± 2.2	18.6 ± 1.8	17.3 ± 1.2	↓ 13.7 ± 1.4*
State 4 mΔΨ	168.9 ± 0.9	↑ 172.9 ± 1.5*	175.1 ± 1.5	↓ 170.4 ± 1.2*
RCR	2.64 ± 0.16	↑ 4.46 ± 0.28***	4.28 ± 0.26	↓ 3.84 ± 0.24*
ADP/O	1.34 ± 0.08	1.42 ± 0.07	2.39 ± 0.11	2.30 ± 0.07

Respiratory rates were measured in the absence (state 4, non-phosphorylating respiration following phosphorylating respiration) or presence (state 3, phosphorylating respiration) of 150 μM ADP. The respiratory control ratios (RCR) are equal to the ratio of state 3 to state 4 respiration. Changes in hypoxia (H) vs. control values (N, normoxia) are marked: increase (↑), decrease (↓)

*P* < 0.05 (*), *P* < 0.01 (**), *P* < 0.001 (***), comparison vs. control value (N); *n* = 15

With the exception of complex I and complex II, the expression of other oxidative phosphorylation system components and CS in mitochondria from hypoxic cells was unaltered (Fig. 1b). Moreover, the maximal COX activity was unaffected (Fig. 5b). However, in mitochondria isolated from hypoxic cells, the oxidation of glutamate was significantly elevated, whereas the oxidation of palmitoylcarnitine was reduced (Fig. 5a). This latter change was accompanied by a downregulation in mitochondrial expression of acyl-CoA dehydrogenase (ACADS), the enzyme that catalyzes the initial step of fatty acid β-oxidation (Fig. 1b).

### Hypoxia-induced changes in mitochondrial ROS formation

In mitochondria isolated from hypoxic endothelial cells, the reduced activity of complex I led to a significant decrease (∼10–14%) in H_2_O_2_ formation during malate oxidation under non-phosphorylating and phosphorylating conditions (Fig. 5c). However, when malate and succinate were oxidized together, a considerable elevation (∼35–45%) of H_2_O_2_ formation was observed in mitochondria from hypoxia-exposed cells. This elevation was caused by the increase in complex II activity resulting in elevated mROS formation involving complex III and mainly complex I-mediated reverse electron transfer. During succinate oxidation in the presence of rotenone (an inhibitor of complex I), a ∼20% increase in H_2_O_2_ formation was detected for non-phosphorylating mitochondria from hypoxic cells. No change was observed with succinate (plus rotenone) under phosphorylating conditions. When succinate was oxidized in the absence of rotenone in mitochondria from hypoxic cells, a greater elevation of H_2_O_2_ formation was observed both under non-phosphorylating (∼50%) and phosphorylating (∼20%) conditions compared to mitochondria from normoxic cells. These results indicate that, in mitochondria, after exposure of endothelial cells to hypoxia, the decreased activity of complex I may produce (i) less ROS when supplied by NAD-linked substrate and (ii) more ROS when reverse electron transport is supplied by upregulated complex II activity.

### Growth under hypoxic conditions induced decreased UCP2 activity

The analysis of UCP2 protein expression (Fig. 1b) showed a slight downregulation (∼20%) of the protein in mitochondria isolated from hypoxic cells relative to those isolated from control cells. No change in the UCP3 expression level was found. Therefore, we attribute the hypoxia-induced changes in UCP activity, described below, to UCP2.

To determine whether UCP2 activity is changed due to the growth of EA.hy926 cells under hypoxic conditions, we evaluated the activation of UCP2 by a free fatty acid (linoleic acid) or a lipid peroxidation product (HNE), and the inhibition by GTP in isolated endothelial mitochondria (Fig. 5d). In general, in non-phosphorylating mitochondria isolated from hypoxic cells, the stimulatory effect of linoleic acid or HNE and the inhibitory effect of GTP were weaker than the effects observed in control mitochondria. An analysis of the proton leak kinetics indicates that for specific linoleic acid (14 μM) and GTP (4 mM) concentrations, the linoleic acid-induced, GTP-inhibited, UCP2-mediated proton leak (UCP2 activity) at the same mΔΨ (162 mV) was ∼18% lower in mitochondria from the hypoxic cells than it was in the control mitochondria (Fig. 5d, left panel). As with linoleic acid, the analysis of the proton leak kinetics indicates that for specific HNE (100 μM) and GTP (4 mM) concentrations, the HNE-induced, GTP-inhibited, UCP2-mediated proton leak at the same mΔΨ (162 mV) was ∼23% lower in mitochondria from the hypoxic cells (Fig. 5d, right panel). Despite slightly decreased UCP2 activity and protein levels, the expression level of superoxide dismutase 2 (SOD2), another mitochondrial antioxidant protein, was unaltered in mitochondria from hypoxic cells (Fig. 1b).

## Discussion

To obtain a sufficient amount of active isolated endothelial mitochondria, we have chosen endothelial EA.hy926 cell line. In vitro cell culture and functional studies with isolated mitochondria are useful in understanding the physiological role that mitochondria play in endothelial cells and the contribution of endothelial mitochondria to hypoxia-induced response. However, there may be some differences in cellular and mitochondrial effects in arteries or in microvascular endothelial cells in vivo.

The hypoxia-induced responses in aerobic metabolism are well documented, for instance, in cancer and brain cells (for reviews see [6, 16, 17, 28]) but this issue has not been intensively studied in endothelial cells. Therefore, the aim of our study was to determine for the first time the effects of chronic hypoxia on mitochondrial oxidative metabolism in endothelial cells, including the effects on isolated endothelial mitochondria. The hypoxia-induced endothelial metabolism changes were supported by a significantly increased level of HIF1-α. The comparison of the mitochondrial respiratory functions of EA.hy926 cells cultured in hypoxic or normoxic conditions demonstrates that deficiency in O_2_ tension induces a general reduction in mitochondrial respiration supplied with carbohydrate catabolic intermediates (glucose or pyruvate), lipid metabolism intermediate (palmitic acid), and amino acids (glutamine, valine, or threonine) whereas cellular respiration with exclusively ketogenic amino acids (lysine or leucine) was significantly increased. The increased oxidation of ketogenic amino acids, which are directly degraded to acetyl-CoA, suggests that the TCA cycle is not impaired in hypoxic endothelial cells in contrast to pyruvate-dependent oxidation. Moreover, in mitochondria from hypoxic EA.hy926 cells, the oxidation of palmitoylcarnitine and the expression of acyl-CoA dehydrogenase were significantly decreased, confirming a hypoxia-induced decrease in fatty acid metabolism. Thus, similar to cancer cells [11], in endothelial cells, hypoxia inhibits fatty acid β-oxidation, another major source of acetyl-CoA. In our study, in mitochondria from hypoxic endothelial cells, the oxidation of pyruvate and the expression of the E3BP component of the pyruvate dehydrogenase complex were unaltered, suggesting that there are other factors downregulating pyruvate-linked oxidation. It has been previously proposed that activation of HIF1-α induces pyruvate dehydrogenase kinase 1 (PDK1), which inhibits the pyruvate dehydrogenase complex and decreases respiration by substrate limitation [13, 21]. The above described metabolic responses observed in this study indicate that this HIF1-α-coordinated regulatory pathway decreasing the TCA cycle activity under hypoxic exposure seems to also occur in endothelial cells. However, limiting mitochondrial fuel oxidation may provide some metabolic advantages to endothelial cells exposed to hypoxia, including the diversion of pyruvate into anabolic pathways and the reduction of apoptosis by hyperpolarized mΔΨ.

In EA.hy926 cells grown for 6 days in hypoxic conditions, the decreased respiration in the presence of glucose was accompanied by elevated expression of hexokinase I and elevated expression and activity of LDH. Thus, in addition to the reduction in aerobic glucose oxidation, endothelial hypoxic cells display enhanced anaerobic glycolysis, which seems to be an important energy source under hypoxic conditions. Our results with endothelial cells are consistent with previous observations from other cell types, namely that hypoxia affects energy metabolism and HIF1-α activates transcription of genes encoding glycolytic enzymes to further increase flux of reducing equivalents from glucose to lactate [18].

It has been well documented that the exposure of endothelial cells to hypoxia leads to increased intracellular ROS production and therefore oxidative stress [4, 22, 24]. Under our experimental conditions, a significant increase in intracellular ROS and mROS generation was also observed. However, the hypoxia-induced oxidative stress does not seem to be excessive for endothelial cells because they maintained cell viability. Moreover, no change in the expression levels of the cytosolic and mitochondrial superoxide dismutases (SOD1 and SOD2, respectively) and UCP2, proteins of the antioxidative systems, was observed. Thus, there was no need for protection against the overwhelming oxidative stress, and increased levels of mROS may be involved in endothelial signaling. Increased mROS are now known to be biologically important in a variety of physiological systems, including adaptation to hypoxia [26, 27]. For instance, cells can utilize an acute increase in mROS to stabilize HIF under hypoxic conditions and subsequently restrain mROS production under chronic hypoxic conditions to avoid cellular damage [26]. Our study indicates that the observed decrease in mitochondrial oxidative metabolism of endothelial cells may help mROS in not exceeding the buffering capacity of superoxide dismutases. Therefore, cell viability of endothelial cells exposed to chronic hypoxia remains unchanged.

Because the activities of COX and CS and the expression level of mitochondrial marker proteins remained unchanged, it appears that the chronic growth of EA.hy926 cells in hypoxia did not change their maximal aerobic respiration capacity or mitochondrial biogenesis. However, our measurements of mitochondrial function in isolated endothelial mitochondria indicate that hypoxia induced important remodeling of the oxidative phosphorylation system at the level of respiratory chain dehydrogenases. In mitochondria from hypoxic endothelial cells, the considerably elevated protein expression and activity of complex II contribute to an increase in mΔΨ and succinate-sustained mROS formation mainly through increased reverse electron transport. It has been previously reported that in endothelial cells, complex II-driven electron flow is the primary means by which the mitochondrial membrane is polarized under hypoxic conditions [10]. Another interesting finding from this study is that, in mitochondria from hypoxic endothelial cells, a reduction of expression and maximal malate-sustained activity of complex I was accompanied by an increase in oxidation of other weaker than malate NAD-linked substrates α-ketoglutarate or isocitrate. Thus, hypoxia-induced reduction of the oxidative phosphorylation system of endothelial mitochondria was found only during malate-sustained complex I activity. Interestingly, it has been reported that lack of oxygen deactivates mitochondrial complex I in many cells [9, 17]. It has also been proposed that hypoxia induces reprogramming of respiratory chain functions and switching from oxidation of complex I substrates to succinate oxidation (complex II) [17, 25]. The dissociation of complex I from the large mitochondrial supercomplexes has been observed under hypoxic conditions, when succinate accumulates as a substrate for complex II [3, 19]. It is well documented that in mammalian cells, hypoxia is connected with activation of succinate dehydrogenase and succinate oxidation and with increased contribution of the latter to respiration and energy production [6, 17, 28]. Under hypoxic conditions, complex II may function as an independent enzyme whose activity is limited only by substrate availability. Hypoxia inhibits the malate-aspartate shuttle, which provides α-ketoglutarate to the cytosol, whereas succinate synthesis is intensified.

We have previously shown that one physiological role of UCP2 in endothelial cells could be the attenuation of mROS production under conditions of excessive oxidative stress, such as exposure to high glucose or palmitic acid concentrations [1, 15]. Results of this study indicate that, in response to hypoxia during endothelial cell growth, UCP2 activity and protein levels were slightly but significantly decreased in mitochondria, even intracellular and mitochondrial ROS formation was increased. Reduction in UCP2 activity leads to higher mΔΨ and consequently increased mROS production. Thus, in hypoxic endothelial cells, one physiological role of UCP2 could be ensuring efficient oxidative phosphorylation yield rather than the attenuation of mROS production.

In mammalian cells, multiple mitochondrial products, including the TCA cycle intermediates and mROS, can coordinate prolyl hydroxylase (PHD) activity and, thereby, HIF stabilization, hence the cellular response to oxygen deficiency [16, 20]. Accumulation of succinate and fumarate inhibits PHDs, leading to an elevated level of HIF, whereas α-ketoglutarate facilitates PHD action, leading to HIF degradation [14]. In our study, in hypoxic endothelial cells, no change in activity of CS, a pace-making enzyme of the first step at the TCA cycle, was observed. However, the observed increase in mitochondrial oxidation of isocitrate, α-ketoglutarate, and glutamate (upstream of succinate oxidation) and the decrease in malate oxidation (downstream of succinate oxidation) could be relevant for maintaining the high level of succinate and fumarate despite the elevated activity of succinate dehydrogenase (complex II). Moreover, significant amelioration of mitochondrial oxidation of α-ketoglutarate may lead to suppression of cytosolic α-ketoglutarate accumulation in endothelial cells exposed to hypoxia. It has been reported that in mammalian cells, especially cancer cells or other highly proliferating cells, ROS, including mROS, are crucial to activating HIF1 [12]. A precise role for mROS in regulating HIF1-α is unclear, but the pathway stabilizing HIF1-α appears undoubtedly mitochondrial dependent [20]. Our study indicates that under hypoxic conditions, endothelial mitochondria also function as an oxygen sensor and convey signals to HIF1 likely through elevated mROS, accumulation of succinate and decreased levels of α-ketoglutarate. Thus, it seems that in endothelial cells, coordinated signaling between HIFs (at least HIF1-α) and the mitochondria regulate the cellular response to chronic hypoxia.

**Fig. 6:**
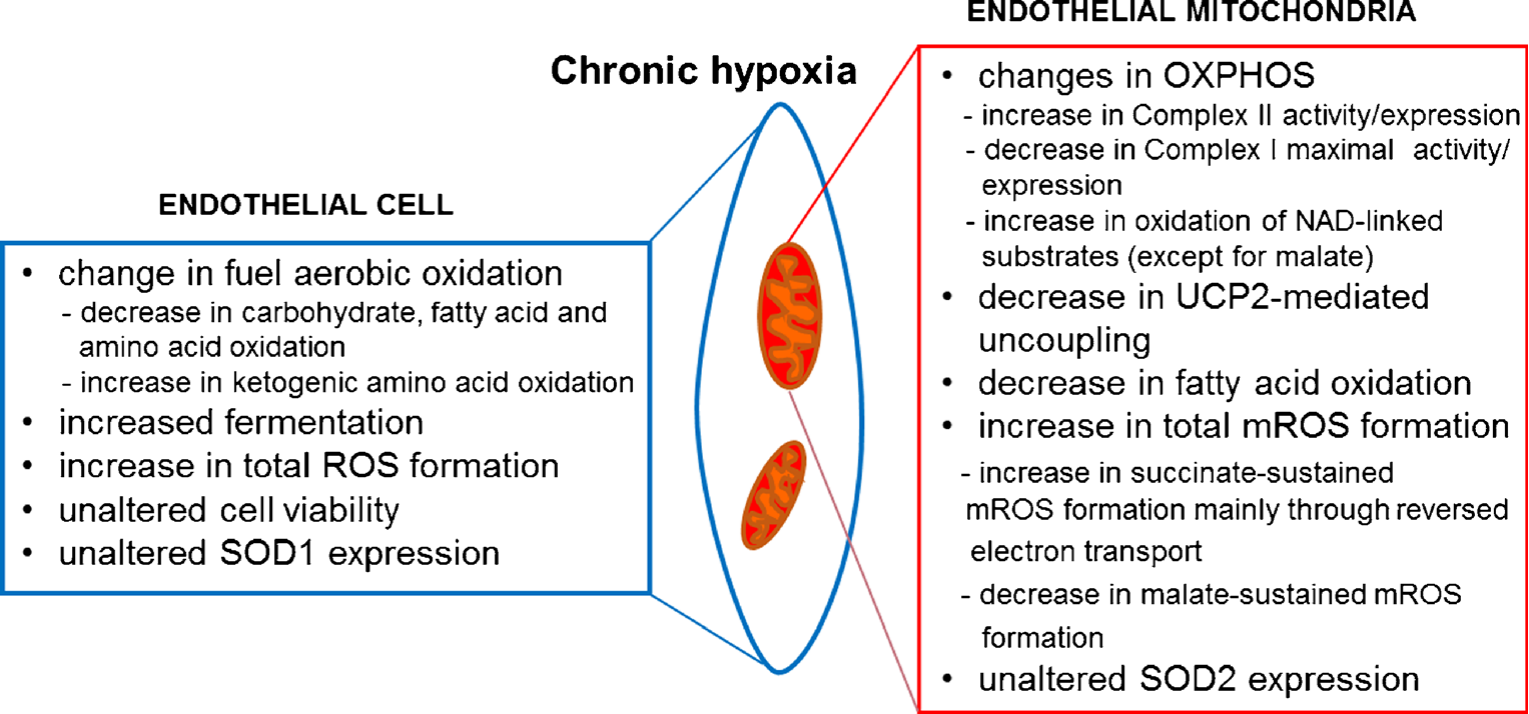
Effects of chronic exposure to hypoxia on endothelial cells and their mitochondria, which are observed in this study. *SOD1* and *SOD2* superoxide dismutases, *OXPHOS* oxidative phosphorylation system

In conclusion, the growth of endothelial cells under chronic hypoxic conditions induces numerous changes in their aerobic metabolism (Fig. 6), particularly a general decrease in mitochondrial respiration except for the increased oxidation of exclusively ketogenic amino acids. The hypoxia-induced increases in intracellular and mitochondrial ROS production do not lead to overwhelming oxidative stress because cell viability and antioxidant systems (superoxide dismutases and UCPs) are not upregulated. The hypoxia-induced increase in mROS formation could result from decreased mitochondrial UCP2-mediated uncoupling and mainly from remodeling of mitochondrial respiratory chain functions with the elevated activity of complex II and decreased activity of complex I. In mitochondria from hypoxic cells, the increased activity of complex II results in an amelioration of succinate-sustained mROS formation mainly through increased reverse electron transport. These observations highlight the role of endothelial mitochondria in response to metabolic adaptations related to hypoxia. The chronic hypoxia-induced decrease in aerobic glucose oxidation may lead to some advantages to endothelial cells, including diversion of pyruvate into anabolic pathways, suppression of apoptosis, preservation of cell proliferation, and activation of the HIF-1, which sustains mitochondrial suppression through increased pyruvate dehydrogenase kinase (PDK) expression. Understanding the details of mitochondrial regulation of chronic hypoxia-induced response in endothelial cells could have clinical relevance and pave the way for new therapies related to hypoxia-associated pathologies.
